# Cervical Tuberculous Lymphadenitis in a Healthy Individual Following Latent Tuberculosis Treatment: A Case Report

**DOI:** 10.1002/rcr2.70216

**Published:** 2025-05-21

**Authors:** Masahiro Yanagi

**Affiliations:** ^1^ Muroran City General Hospital Department of Respiratory Medicine Muroran Japan

**Keywords:** cervical tuberculous lymphadenitis, extrapulmonary tuberculosis, latent tuberculosis infection, *Mycobacterium tuberculosis*

## Abstract

A 33‐year‐old healthy female, employed as a hospital‐based physical therapist, was exposed to a colleague diagnosed with pulmonary tuberculosis. Screening revealed a positive interferon‐gamma release assay (IGRA) result. She was diagnosed with latent tuberculosis infection (LTBI) and completed a 6‐month isoniazid (INH) regimen. Nine months post‐treatment, she presented to the otolaryngology department with right cervical swelling and ulceration. Polymerase chain reaction (PCR) of the pus confirmed *Mycobacterium tuberculosis* , establishing a diagnosis of cervical tuberculous lymphadenitis. She was referred to our hospital, where anti‐tuberculosis therapy led to symptom resolution. Post‐LTBI follow‐up primarily monitors for pulmonary tuberculosis, focusing on chest abnormalities or respiratory symptoms, particularly in high‐risk individuals with immunodeficiency. However, extrapulmonary tuberculosis can occur even in healthy individual's post‐LTBI treatment, necessitating vigilance.

## Graphical abstract

We present a case of cervical tuberculous lymphadenitis in a healthy individual following latent tuberculosis infection (LTBI) treatment.

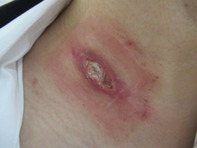

## 1 Introduction

Latent tuberculosis infection (LTBI) treatment effectively reduces the risk of progression to active tuberculosis (TB) and prevents further disease transmission. Despite treatment, TB may still develop, particularly in individuals with immunodeficiencies or underlying conditions necessitating closer monitoring. Post‐LTBI follow‐up typically focuses on pulmonary TB surveillance, including chest x‐rays and respiratory symptom assessment. However, in rare cases, TB manifests as extrapulmonary disease with atypical presentations. We present a case of cervical tuberculous lymphadenitis in a healthy individual following LTBI treatment.

## 2 Case Report

**FIGURE 1:**
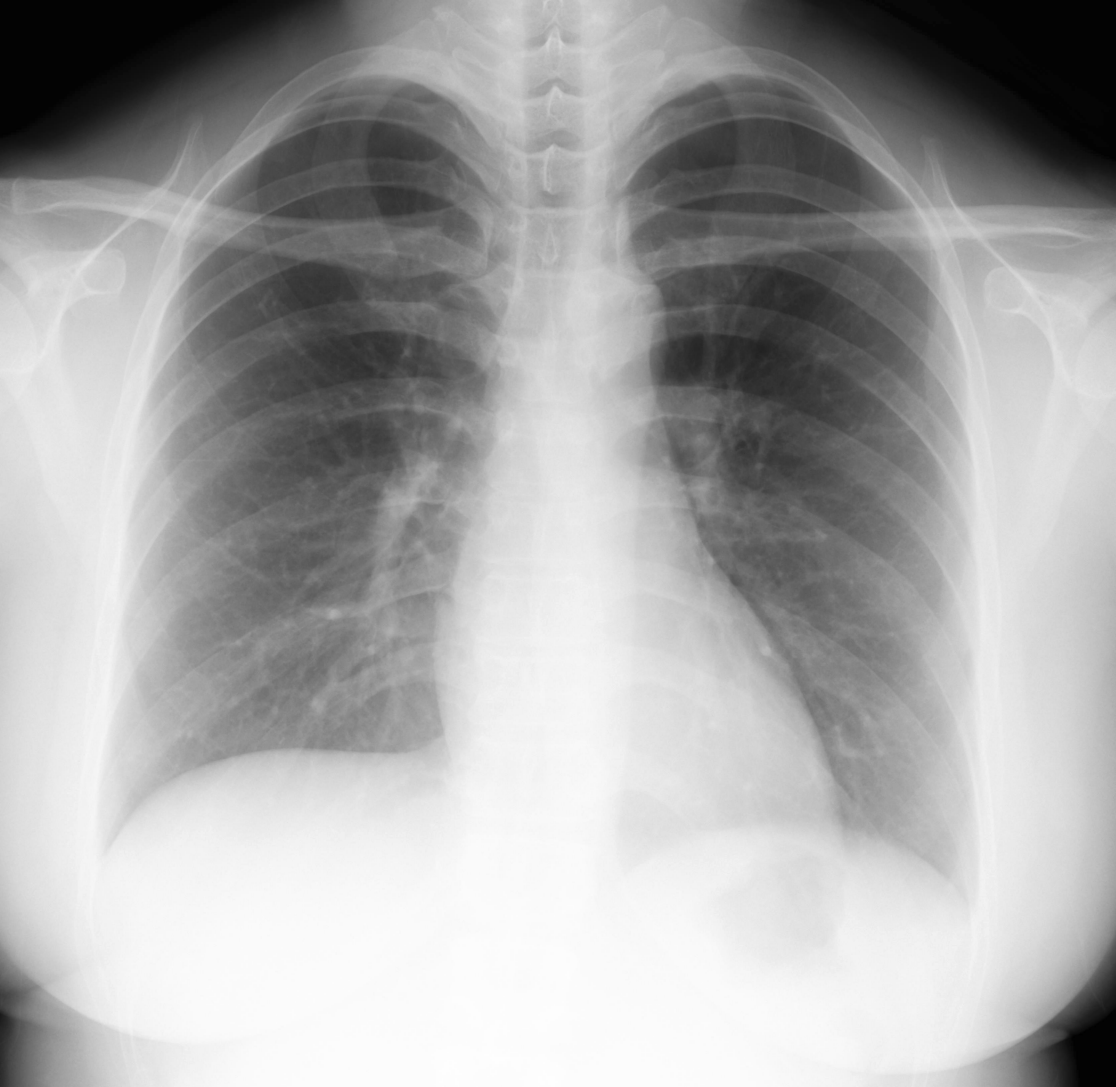
The chest x‐ray shows no abnormalities.

A 33‐year‐old female with no significant medical history worked as a physical therapist at a hospital and was considered a close contact after a colleague was diagnosed with smear‐positive pulmonary TB. Subsequent screening revealed a positive interferon‐gamma release assay (IGRA) result. She was asymptomatic, with no lymphadenopathy on physical exam. Chest x‐ray (Figure 1) was normal, and she was diagnosed with LTBI. She completed a six‐month isoniazid (INH, 300 mg/day) course with adherence confirmed via directly observed treatment short‐course (DOTS) by the hospital and public health centre. After treatment, she developed no diabetes, autoimmune diseases or immunodeficiency‐related infections, and no further follow‐up was conducted. However, 9 months later, she presented to otolaryngology with right cervical swelling and ulceration.

**FIGURE 2:**
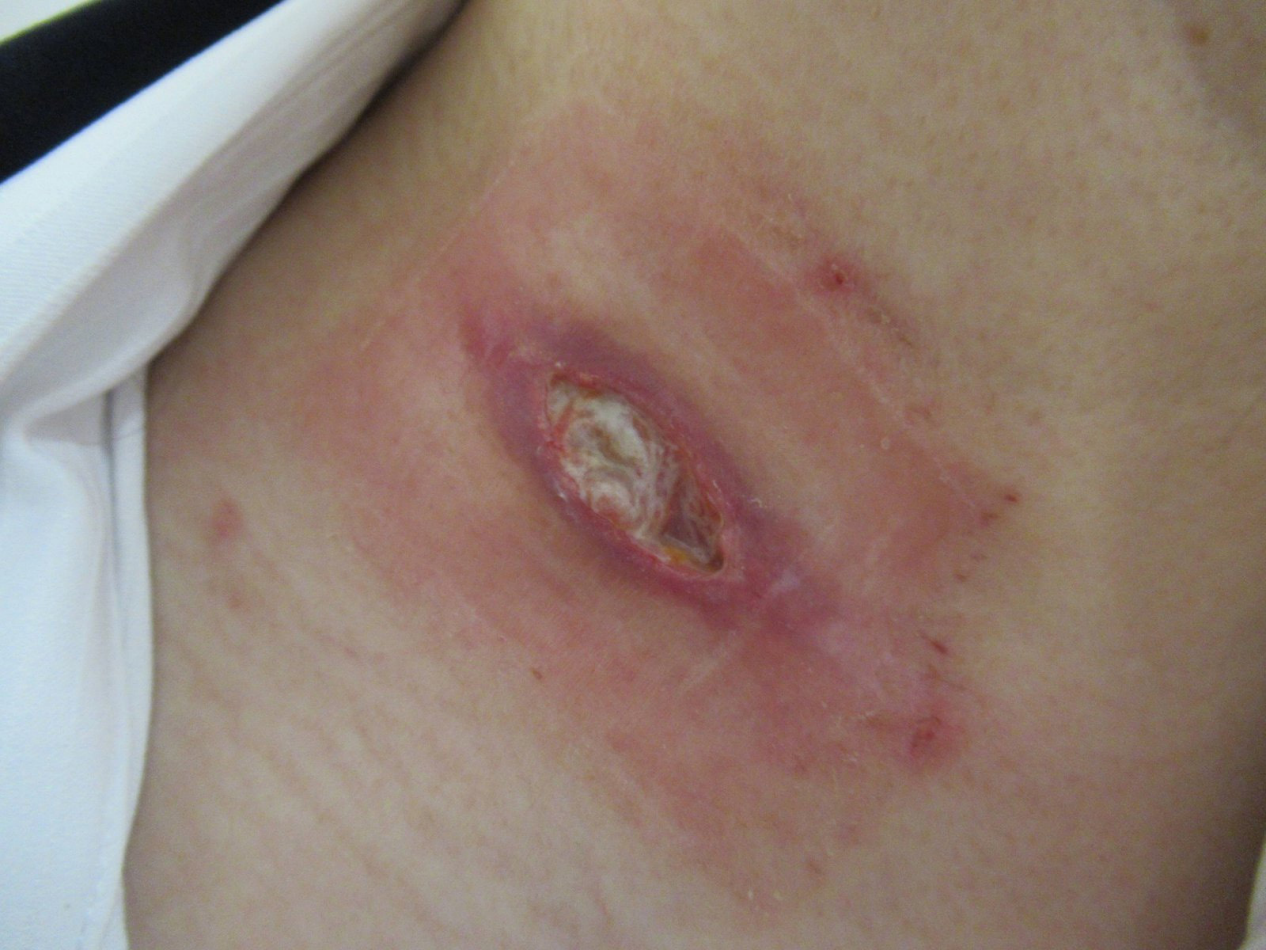
Swelling, redness and ulcer formation are observed in the right cervical region.

**FIGURE 3:**
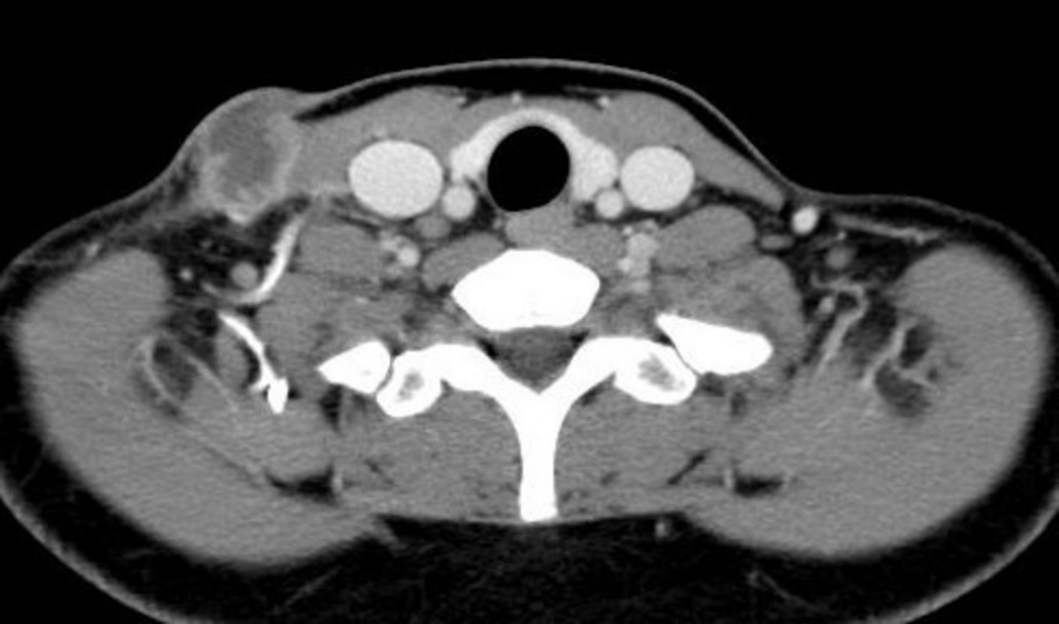
Contrast‐enhanced CT showing an enlarged right cervical lymph node with low internal attenuation.

A 2 cm, soft, mobile lymph node swelling was observed in the right cervical region, with surrounding erythema and ulcer formation (Figure 2). She exhibited no systemic symptoms, including fever, fatigue, night sweats, or weight loss. Laboratory tests revealed normal inflammatory (total white blood cell count: 6690/μL; C‐reactive protein: 0.04 mg/dL) and tumour markers, along with a negative HIV antibody test. Contrast‐enhanced CT imaging revealed a 28 × 21 mm right cervical lymph node with low internal attenuation (Figure 3) and a similarly attenuated 10 mm prevascular lymph node (#3a) in the anterior mediastinum. The exudate's polymer chain reaction (PCR) confirmed *Mycobacterium tuberculosis* , establishing a diagnosis of cervical tuberculous lymphadenitis. No mycobacteria were cultured, and drug susceptibility testing was not performed. GeneXpert MTB/RIF was not performed on clinical specimens. Apart from these lesions, no other abnormalities, including pulmonary involvement, were detected. Additionally, sputum smear and culture for acid‐fast bacilli were negative. As surgery was not a viable option, she was referred to our department for pharmacologic management.

**FIGURE 4:**
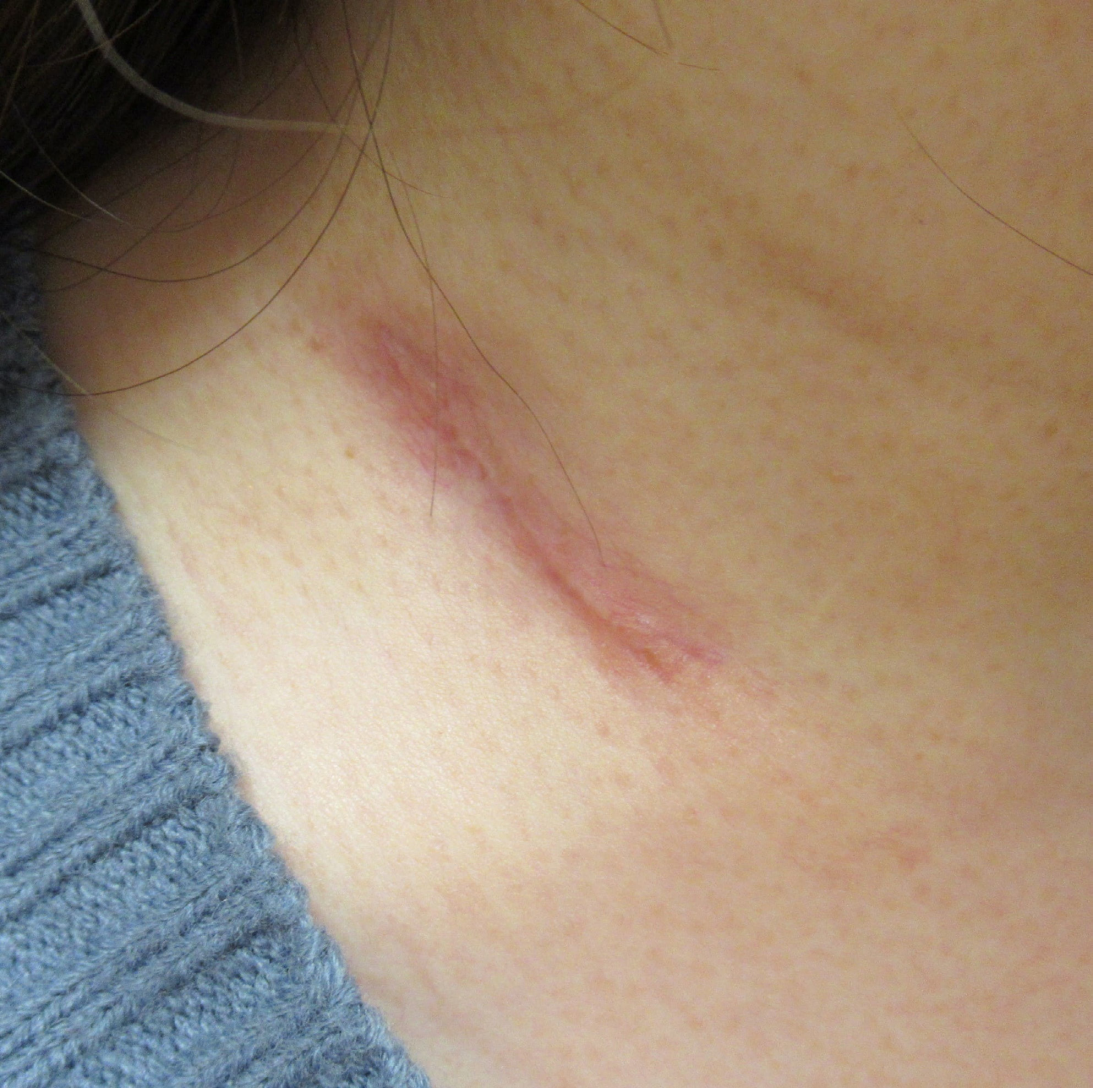
The swelling and ulcer in the right cervical region improved.

**FIGURE 5:**
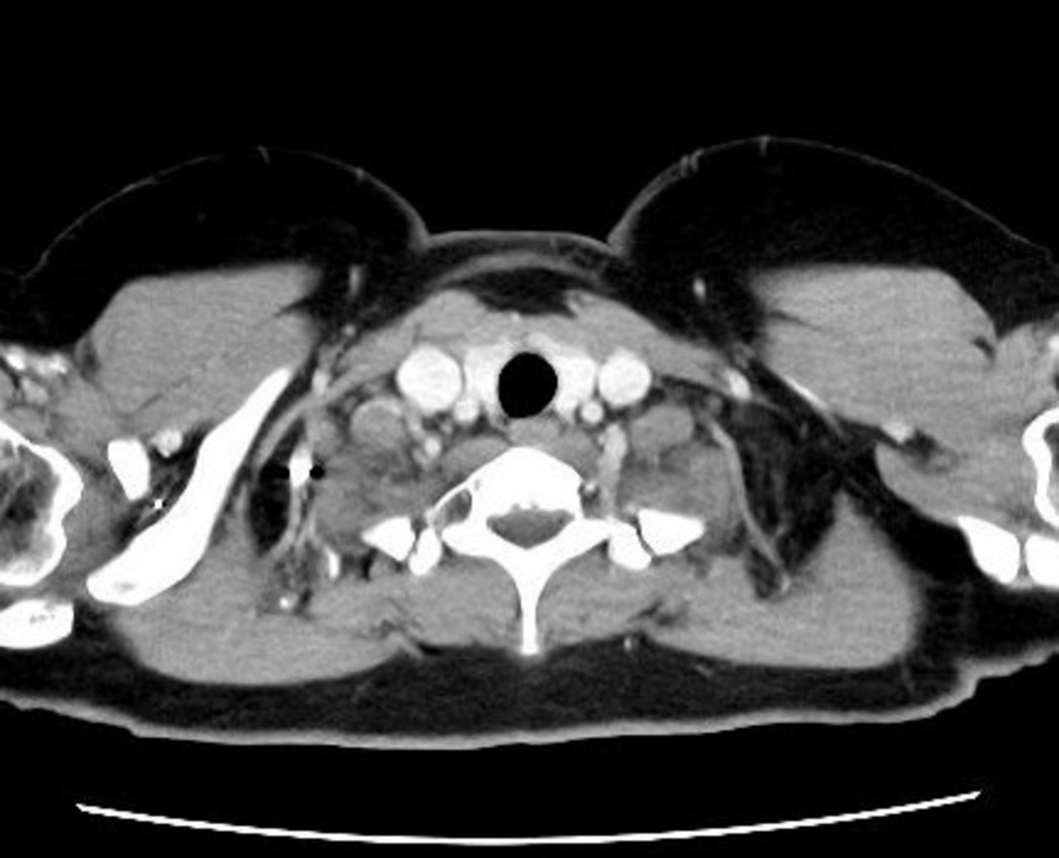
Contrast‐enhanced CT showed resolution of the right cervical lymph node.

She was initiated on a four‐drug regimen of INH, rifampicin (RFP), ethambutol (EB), and pyrazinamide (PZA). Due to PZA‐induced liver dysfunction, the regimen was adjusted to an INH, RFP, and EB for 2 months, followed by a seven‐month maintenance therapy with INH and RFP. Fixed‐dose combination drugs were not used. The DOTS strategy was implemented for TB treatment. The right cervical swelling gradually subsided and nearly resolved by the seventh month of therapy (Figure 4). Furthermore, follow‐up CT imaging showed near resolution of the lymph nodes (Figure 5).

## 3 Discussion

LTBI treatment was introduced to reduce the risk of progression to active TB, highlighting the importance of early intervention [1]. The widespread adoption of IGRA has recently enhanced the early diagnosis and management of LTBI. WHO guidelines recommend multiple LTBI treatment regimens, including six‐ or nine‐month courses of INH, 3 to 4 months of RFP, or combination therapy with INH and RFP for 3 to 4 months [2]. Each reduces TB risk by ~50%–70%, with generally equivalent effectiveness. INH is widely available but carries a risk of hepatotoxicity. RFP, used alone or in combination, offers shorter treatment durations but requires caution due to drug interactions. Additional concerns include its higher cost and lack of approval in some countries [1, 2]. In Japan, RFP is increasingly used when INH is contraindicated, though many facilities still favour INH. An analysis of LTBI treatment involving over 20,000 individuals in Istanbul reported a post‐treatment TB incidence rate of 0.5%, indicating that active TB development after LTBI treatment is rare [3]. However, with a treatment completion rate of ~60% [3], current LTBI regimens may have limitations related to side effects and adherence, hindering successful therapy completion.

Despite LTBI treatment, TB may still develop in some individuals, requiring ongoing follow‐up and patient education. In Japan, individuals treated for LTBI must undergo regular monitoring for 2 years, mainly via chest x‐rays, under the Infectious Diseases Act. Many facilities conduct follow‐ups every 6 months. However, based on adherence and group infection rates, follow‐up may be waived for those at low risk. However, regional differences in hospital and public health centre resources mean follow‐up systems are not standardised. Given limited resources, a system to promote patient education—such as distributing informational pamphlets—is needed to raise awareness of recurrence risk after LTBI treatment. A retrospective analysis in Japan of LTBI patients registered between 2008 and 2009 (*n* = 8951) found that 56 individuals developed active TB by 2011 [4]. The incidence of active TB within 2 years of LTBI treatment was 0.57% (51/8951) [4]. Of the 56 patients who developed TB, 45 had pulmonary lesions, three had isolated pleuritis and eight had lymph node TB without pulmonary involvement. However, the overall health status and underlying conditions of patients who developed TB remain unknown [4]. This case of lymph node TB in a young, immunocompetent individual post‐LTBI treatment is rare.

Extrapulmonary TB, including lymphadenitis, accounts for ~15%–20% of all TB cases, with cervical lymph nodes being the most frequently affected. Unlike pulmonary TB, which predominantly affects older men, cervical TB is more common in younger individuals and females [5, 6]. Skin fistula formation occurs in approximately 10% of cervical TB lymphadenitis cases [5]. Some cases present without systemic symptoms such as fever, with cervical swelling as the sole complaint, complicating diagnosis [6]. Residual lymph nodes may persist in 15%–30% of patients post‐treatment and do not necessarily indicate treatment failure [6]. Post‐LTBI treatment surveillance typically focuses on detecting pulmonary TB via chest imaging abnormalities or respiratory symptoms. However, given the risk of extrapulmonary TB, patients should be informed of its atypical presentations.

Mycobacterial culture results were unavailable in this case, preventing drug susceptibility testing. However, a review of the source patient's drug susceptibility test revealed no resistance to anti‐TB medications. Despite this, cases of INH‐resistant TB have been reported following LTBI treatment [4]. Although no evidence suggests that LTBI treatment with INH contributes to INH resistance [2], every effort should be made to obtain culture specimens in TB onset post‐LTBI treatment.

Host factors contributing to LTBI progression to active TB include HIV, low body weight, diabetes, chronic renal failure, organ transplantation, fibrotic chest x‐ray lesions (indicative of prior untreated TB), silicosis, and cancer [1]. In young individuals, assessing HIV, diabetes and autoimmune diseases is crucial. Furthermore, evaluating medication adherence, *Mycobacterium tuberculosis* exposure extent and duration, and drug resistance helps estimate recurrence risk. Comprehensive consideration of these factors improves treatment success and prevents missed diagnoses in recurrent cases. Healthcare workers, as in the present case, may face repeated exposure and pose a transmission risk if they develop TB, requiring closer monitoring. A follow‐up strategy for high‐risk populations is reasonable, considering human resources and cost‐effectiveness. However, as demonstrated in this case, TB may also occur in healthy individuals. Although rare, extrapulmonary TB remains a possibility and warrants clinical vigilance.

## Author Contributions

The author was responsible for the study's conceptualisation, design, data acquisition, analysis and interpretation.

## Consent

The author declares that written informed consent was obtained for the publication of this manuscript and accompanying images using the consent form provided by the Journal.

## Conflicts of Interest

The author declares no conflicts of interest.

## Data Availability

The data that support the findings of this study are available on request from the corresponding author. The data are not publicly available due to privacy or ethical restrictions.
